# Multistage Reconstruction of a Severe Cloacal Exstrophy Variant With Associated Myelomeningocele and Multisystem Involvement: A Case Report

**DOI:** 10.7759/cureus.106847

**Published:** 2026-04-11

**Authors:** Mustapha Akhdar, Abd El Rahman Abd El Barr

**Affiliations:** 1 Surgery, AdventHealth Florida, Tampa, USA; 2 Pediatric Urology, AdventHealth Florida, Tampa, USA

**Keywords:** cloacal exstrophy, functional urology, myelomeningocele (mmc), omphalocele, pediatric urology surgery, pelvic reconstructive surgery, plastic and reconstructive surgery, reconstructive, reconstructive surgery, urology

## Abstract

To our knowledge, this case represents one of the rarest and most severe forms of cloacal exstrophy (CE), distinguished by extensive multisystem involvement, including a large omphalocele, imperforate anus, bladder exstrophy, bilateral clubfeet, and the unusual concomitant presence of a lumbosacral myelomeningocele. This constellation of findings is exceptionally uncommon and poses significant surgical and multidisciplinary challenges from birth.

Prenatal MRI demonstrated a large omphalocele containing liver, bowel, and gallbladder; a lumbar myelomeningocele (L5-S5); nonvisualization of the bladder and external genitalia; unilateral renal agenesis; and bilateral clubfeet. The infant was delivered at 37 weeks via cesarean section and required intubation at 10 minutes of life. Postnatal evaluation confirmed omphalocele, imperforate anus, meningocele, bladder exstrophy, and bilateral clubfeet. Echocardiography revealed a small mid-muscular ventricular septal defect and a moderate patent ductus arteriosus with bidirectional shunt, requiring no intervention. SNP microarray testing was negative for copy number abnormalities.

At five days of life, the patient underwent excision of the omphalocele, colostomy creation, colon conduit cystoplasty, and first-stage closure of the bladder plate. At four months, neurosurgical repair of the lipomeningocele with resection of an extradural lipoma, bilateral muscle flaps, and complex soft-tissue reconstruction was performed. Orthopedic management included treatment of a right leg fracture, followed by bilateral pelvic osteotomies and application of a hip spica cast at eleven months. Definitive bladder exstrophy closure was achieved after orthopedic stabilization. The patient demonstrated stable cardiopulmonary status, preserved renal function, and appropriate recovery following each stage of repair.

This case illustrates the extreme end of the CE spectrum and underscores the necessity of multidisciplinary coordination among pediatric surgery, urology, neurosurgery, orthopedics, and plastic surgery. Through careful sequencing and staged reconstruction, preservation of gastrointestinal, neurologic, orthopedic, and urologic function was achieved. Our experience highlights that, even in the most severe and rare presentations, particularly those with concurrent myelomeningocele, functional survival and meaningful quality of life are possible with meticulous planning and comprehensive care.

## Introduction

Cloacal exstrophy (CE) is an extremely rare and severe congenital malformation within the exstrophy-epispadias complex, characterized by a combination of urinary bladder exstrophy, a prolapsed cecal plate, imperforate anus, omphalocele, and complex genital anomalies. Additional anomalies involving the spine, kidneys, and musculoskeletal system are frequently observed, contributing to the significant morbidity associated with this condition. CE remains an exceptionally rare congenital malformation, with an estimated incidence of 1 in 200,000-400,000 live births [1]. Despite advances in prenatal detection and neonatal care, its overall prevalence remains low. A large single-institution study from the United States reported only 145 cases over a nearly 50-year period (1974-2023) [1].

Historically, CE was considered a universally fatal diagnosis, with most affected infants succumbing during the neonatal period due to sepsis, renal failure, gastrointestinal complications, and neurologic sequelae. The first documented case of long-term survival following a three-stage operative intervention was reported in 1960 [2], marking a pivotal shift in management. Since then, advances in neonatal care, surgical techniques, and multidisciplinary management have dramatically improved survival, and today, most patients with CE survive into adulthood.

Despite these improvements, CE remains associated with significant long-term morbidity. Patients with severe variants often face challenges including urinary and fecal incontinence, impaired renal function, growth failure, musculoskeletal deformities, and limitations in mobility. Functional outcomes vary depending on the extent of anomalies and the timing and type of surgical interventions. The choice between single-stage versus multistage repair remains a topic of ongoing discussion; while single-stage reconstruction may be feasible in select cases with favorable anatomy, multistage repair is generally favored for optimizing closure success and reducing complications in complex presentations [2].

Modern management emphasizes individualized, multidisciplinary care focused not only on survival but also on improving quality of life. This includes achieving social continence through urinary and fecal reconstruction, preserving renal function, addressing spinal and orthopedic anomalies, and supporting psychosocial development. Understanding the evolving prognosis, long-term functional outcomes, and surgical strategies is essential for counseling families and planning comprehensive care for patients with this challenging condition. To our knowledge, reports describing CE with concurrent large omphalocele and myelomeningocele managed successfully through staged reconstruction remain exceedingly limited.

## Case presentation

**Figure 1:**
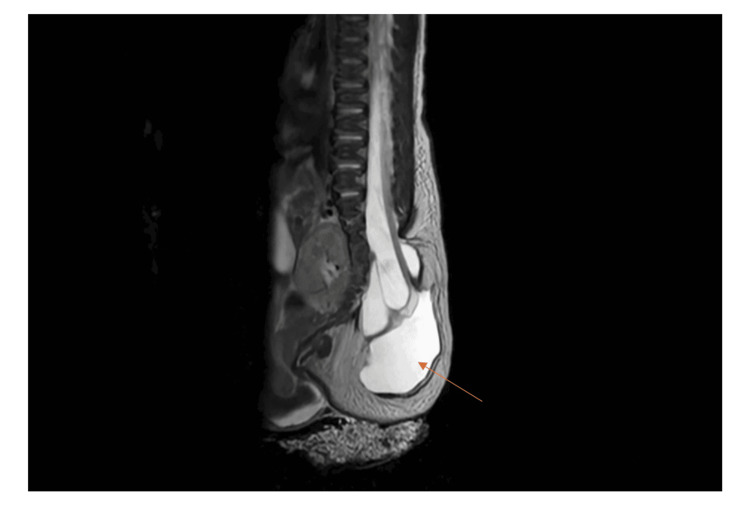
Sagittal-view T2 MRI of myelomeningocele (red arrow)

A 30-week gestational age fetus was evaluated with prenatal ultrasound and non-contrast fetal MRI, which revealed findings consistent with the OEIS (Omphalocele, Exstrophy of the Cloaca, Imperforate Anus, and Spinal Defects) complex. The prenatal imaging demonstrated a large omphalocele measuring approximately 5.5 × 4 × 6.6 cm that contained the liver, multiple bowel loops, and possibly the gallbladder. The urinary bladder and external genitalia were not visualized, findings that were highly suggestive of bladder exstrophy. Additionally, there was evidence of severe pelvic dysplasia. The spinal evaluation revealed a large covered myelomeningocele extending from L5 to S5, involving neural tissue with associated splaying of the posterior elements (Figure 1). Examination of the extremities showed bilateral clubfeet, while the upper extremities appeared normal. The brain demonstrated normal sulcation and structure without evidence of a Chiari II malformation. The right kidney was visualized and measured 3.7 cm in length, while the left kidney was not clearly identified. The umbilical cord contained three vessels, and both the placenta and maternal anatomy appeared normal. Fetal genetic testing using single-nucleotide polymorphism (SNP) chromosomal microarray revealed no copy number abnormalities. The family received multidisciplinary prenatal counseling regarding prognosis and staged surgical options.

The infant was delivered via cesarean section at 37 weeks and 1 day of gestation, due to prenatal concerns regarding the large omphalocele and spinal lesion. At delivery, initial resuscitation was required, including intubation at 10 minutes of life for acute respiratory distress. The Apgar scores were 4 at one minute and 9 at five minutes. The newborn’s birth weight was 3290 grams (7 pounds, 4.1 ounces). Postnatally, the diagnosis of OEIS complex was confirmed, consisting of a large omphalocele, imperforate anus, covered myelomeningocele, bladder exstrophy, and bilateral clubfeet.

On initial physical examination, the infant exhibited a large anterior abdominal wall defect with exposed liver and bowel consistent with the omphalocele, bifid bladder plates indicative of bladder exstrophy, a lumbosacral cystic mass consistent with myelomeningocele, and bilateral talipes equinovarus deformities. Vital signs and laboratory values remained within expected neonatal parameters after stabilization. Subsequent imaging and specialist evaluations were conducted as part of multidisciplinary management. Echocardiography performed by pediatric cardiology revealed a tiny mid-muscular ventricular septal defect and a moderate patent ductus arteriosus with bidirectional shunting; both lesions were hemodynamically insignificant and did not require intervention (Video 1). MRI of the spine confirmed the diagnosis of myelomeningocele without additional intracranial anomalies. Genetic testing reaffirmed normal chromosomal findings with a negative SNP microarray. Orthopedic evaluation was performed for limb deformities, including the bilateral clubfeet, and plans for future corrective management were discussed.

**Video 1:** Echocardiography demonstrating moderate VSD and PDA VSD: Ventricular septal defect; PDA: patent ductus arteriosus

Multidisciplinary surgical management

A staged surgical approach was selected, given the extensive omphalocele with hepatic herniation, CE, and associated multisystem anomalies. Attempting complete neonatal closure would have posed a high risk of abdominal compartment syndrome, wound dehiscence, and compromised tissue perfusion. Therefore, reconstruction was planned in sequential stages to allow physiologic stabilization, tissue maturation, and optimization of outcomes (Table 1). This strategy aligns with contemporary staged protocols for complex CE described in recent literature.

**Table 1:** Timeline of staged interventions for OEIS complex OEIS: Omphalocele, Exstrophy of the Cloaca, Imperforate Anus, and Spinal Defects

Patient Age	Procedure/Intervention	Specialty	Key Notes/Challenges
Birth (37w1d)	Delivery via C-section; intubation at 10 min	Neonatology	Acute respiratory distress; initial stabilization
5 days	Excision of omphalocele, creation of colostomy, colon conduit cystoplasty, first-stage bladder exstrophy closure	Pediatric Surgery/Urology	Large omphalocele with liver involvement; staged bladder repair to minimize tension and infection risk
4 months	Repair of lipomeningocele, resection of extradural lipoma, bilateral muscle & fasciocutaneous flaps, local tissue rearrangement	Neurosurgery/Plastic Surgery	Complex closure of myelomeningocele; tissue coverage challenges; careful flap planning
5 months	Management of right leg fracture (nonoperative)	Orthopedics	Healing monitored in spica cast; no surgery required
11 months	Bilateral pelvic osteotomy, application of two-leg hip spica cast	Orthopedics	Correction of severe pelvic dysplasia; positioning critical for hip stability
11 months	Definitive bladder exstrophy closure	Urology/Pediatric Surgery	Final bladder closure with flap reconstruction; careful monitoring to prevent wound complications

Stage I consisted of neonatal stabilization and initial reconstruction, including excision of the omphalocele membrane, reduction of herniated viscera, tubularization of the cecal plate into a colon conduit, and creation of an end colostomy. Partial approximation of the bladder plates was performed to reduce exposure while avoiding excessive tension. This approach provided protection of abdominal contents, urinary diversion, and controlled gastrointestinal decompression while minimizing the risk of abdominal compartment syndrome. The patient tolerated the procedure well and was extubated postoperatively without complication.

Stage II involved repair of the lumbosacral myelomeningocele in collaboration with pediatric neurosurgery and plastic surgery. The spinal cord was detethered, and the dural defect was closed in a watertight fashion. Bilateral paraspinal muscle flaps and local soft-tissue rearrangement were utilized to provide robust coverage of the neural repair and achieve tension-free closure. Postoperatively, the patient was managed in prone and lateral positioning to protect the repair, with no evidence of cerebrospinal fluid leak or wound complication.

**Figure 2:**
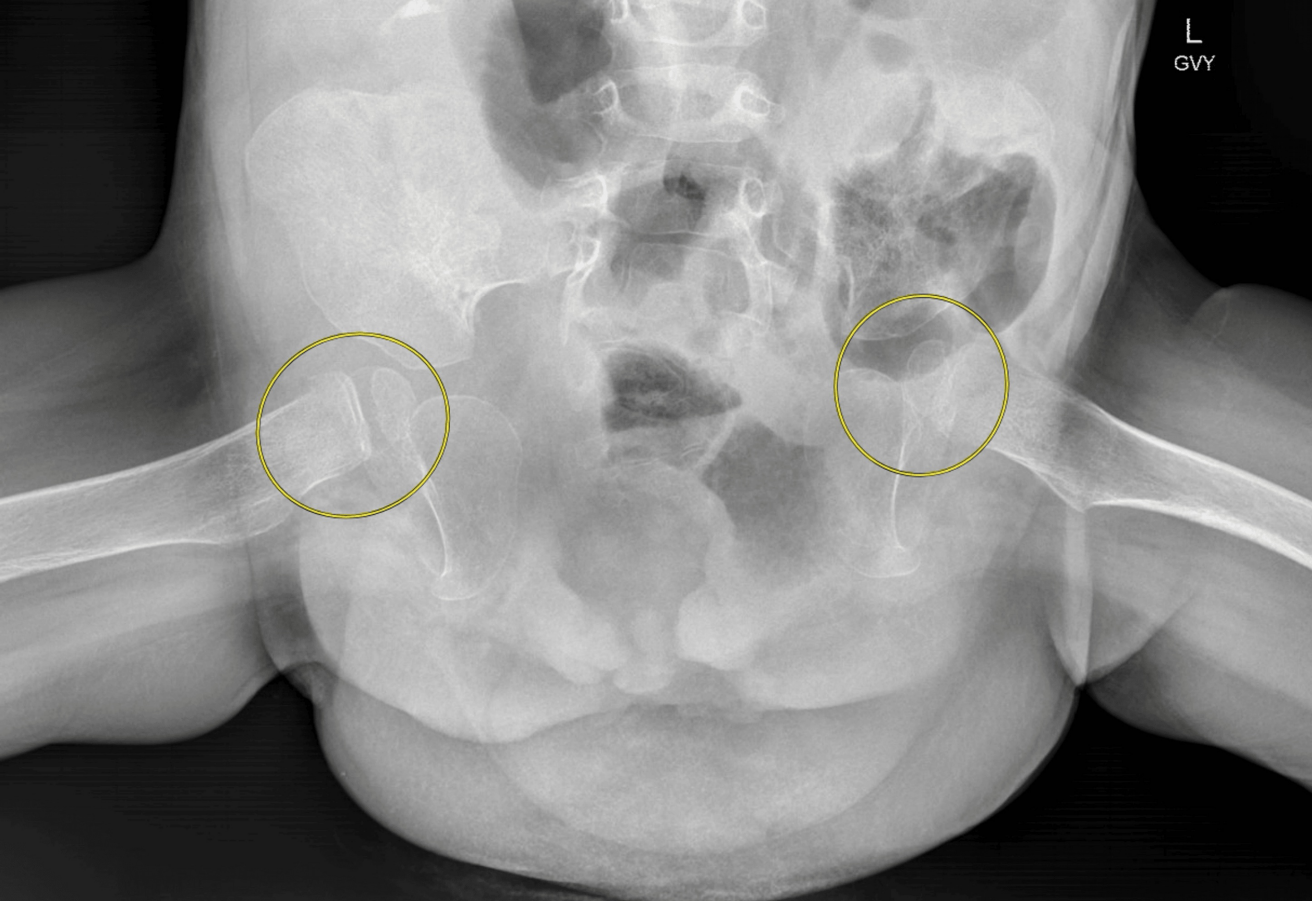
Pelvic X-ray after bilateral pelvic osteotomies (yellow circles)

Stage III consisted of bilateral pelvic osteotomies (Figure 2) performed by pediatric orthopedics to facilitate pelvic mobility and enable subsequent bladder and abdominal wall reconstruction. Following osteotomy, a customized two-leg hip spica cast was applied to maintain alignment and stability. The cast was modified into a removable two-shell design to allow access to the bladder exstrophy site for ongoing care while preserving pelvic immobilization. This orthopedic intervention was essential in reducing tension during definitive closure.

**Figure 3:**
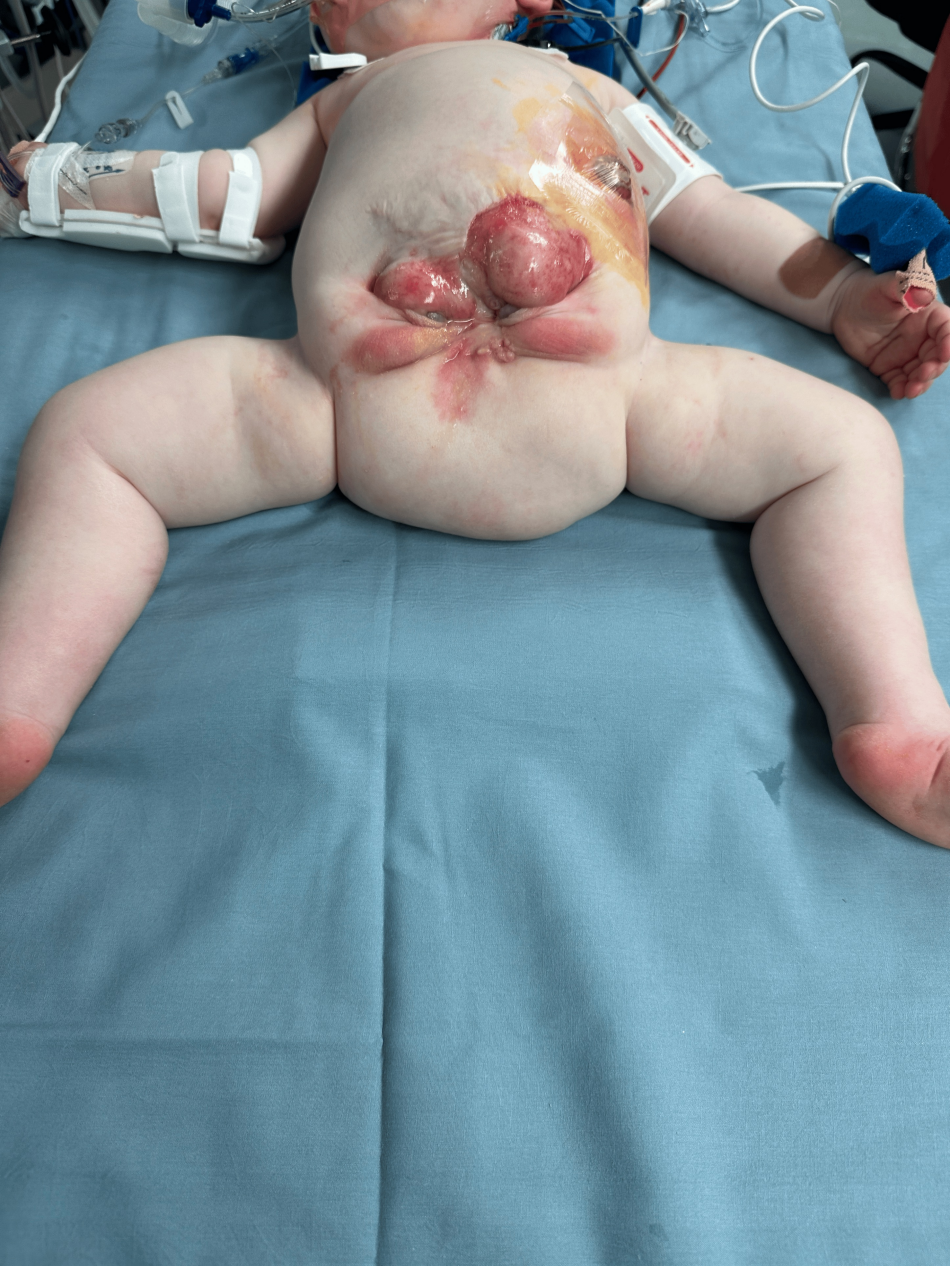
Before definitive closure of bladder exstrophy

Stage IV involved definitive bladder reconstruction and abdominal wall closure, performed after pelvic realignment. After removal of the hip spica cast, the hemibladders were clearly visualized (Figure 3). The hemibladders were fully mobilized and reconstructed into a unified reservoir, which was pexed to the sacral promontory to reduce the risk of prolapse. Abdominal wall closure was achieved using mobilized native fascia reinforced with biologic pericardial grafts in areas of attenuation. This reinforcement allowed tension-free closure while reducing the risk of dehiscence or ventral hernia. The procedure was completed without an intraoperative complication, and the patient recovered uneventfully.

The staged reconstructive strategy allowed for physiologic stabilization, protection of exposed viscera, and gradual correction of pelvic and abdominal anatomy. Pelvic osteotomies were deliberately timed prior to definitive bladder closure to facilitate approximation of the pubic bones and minimize tension on the repair. The use of biologic graft material reinforced attenuated fascia, supporting durable closure in the setting of congenital tissue deficiency and prior surgeries.

Results

The patient tolerated all four stages of reconstruction without major intraoperative or immediate postoperative complications. Postoperatively, he remained hemodynamically stable with no evidence of infection, wound dehiscence, or cerebrospinal fluid leak. The omphalocele repair and bladder plate approximation maintained tissue integrity, and the colon conduit functioned appropriately, allowing for controlled fecal diversion.

Following myelomeningocele repair, neurological assessment demonstrated intact lower extremity motor and sensory function appropriate for age. No signs of tethered cord or CSF leakage were noted during follow-up. Orthopedic intervention with bilateral pelvic osteotomies and custom two-shell hip spica casting provided adequate pelvic alignment, facilitating tension-free closure and supporting mobility.

At the last follow-up (age 12 months), the patient demonstrated appropriate growth for age, stable renal function with a visualized solitary right kidney, and patent urinary and fecal diversions. Bilateral clubfoot deformities were adequately addressed with orthopedic bracing and serial follow-up. The bladder reconstruction was intact, and early postoperative imaging confirmed anatomic alignment without evidence of prolapse or obstruction. Nutritional status was appropriate, and the patient achieved developmental milestones for age.

Overall, the staged, multidisciplinary approach resulted in successful stabilization and functional preservation across multiple organ systems. The combination of neonatal stabilization, sequential surgical correction, orthopedic facilitation, and biologically reinforced abdominal wall closure allowed for progressive anatomical reconstruction while minimizing physiologic stress.

## Discussion

CE represents the most severe spectrum of the exstrophy-epispadias complex and remains a formidable reconstructive challenge due to simultaneous involvement of genitourinary, gastrointestinal, neurologic, and musculoskeletal systems. Historically considered universally fatal, modern advances in neonatal care, surgical techniques, and multidisciplinary coordination have dramatically improved survival, with contemporary series reporting survival rates exceeding 90% [2,3].

Management strategies for CE fall broadly into single‑stage and multistage reconstructions. Single‑stage repair aims to address abdominal wall closure, urinary reconstruction, and genitourinary alignment in one operation. While feasible in selected cases with favorable anatomy, several studies highlight limitations of this approach, including higher rates of failed primary closure, increased wound complications, and technical challenges related to tension and compromised tissue perfusion [4].

In contrast, multistage approaches, beginning with neonatal stabilization, delayed bladder closure, and staged orthopedic management, have shown superior outcomes in many cases of complex CE. A large retrospective study demonstrated that the odds of successful primary bladder closure were significantly higher with a staged approach compared with a one‑stage repair, and that inclusion of pelvic osteotomy further increased success rates. Similarly, the dual‑staged pathway, incorporating staged osteotomy and bladder closure, produced consistent closure success rates even in patients with large diastasis and complex anatomy. Early descriptions of staged pelvic closure also emphasize excellent functional and cosmetic results, supporting its use when primary closure is unfeasible [5-7].

Comparative studies in diverse settings, including low‑ and middle‑income countries, suggest that individualization of surgical strategy is critical, with staged repair favored for patients with larger pubic diastasis, lower birth weight, or significant comorbidities [8,9]. Although long‑term continence outcomes remain variable irrespective of the reconstruction strategy, staged repair consistently mitigates early closure failure and facilitates reconstruction in anatomically complex patients.

Our case exemplifies the rationale for a multistage, multidisciplinary approach. Given the extreme presentation - including large omphalocele, myelomeningocele, and significant pelvic dysplasia - neonatal stabilization followed by sequential orthopedic facilitation, neurosurgical repair, and definitive bladder reconstruction provided safe anatomic correction while minimizing physiologic stress. The successful anatomical and functional outcomes in this patient reinforce contemporary literature supporting staged management in severe CE variants. Moreover, our use of biologic fascial reinforcement adds an additional strategy aligned with reports advocating adjunctive materials to support tension‑free closure. 

Long‑term functional studies in larger CE cohorts demonstrate that continence and bladder management often require additional interventions, regardless of initial surgical strategy. A cross‑institutional study of 160 patients reported that most older children and adults require catheterizable channels or incontinent diversion, highlighting that establishing functional goals early and counseling families realistically are essential components of care [10,11]. Future studies should continue to define long‑term functional outcomes, including urinary and bowel continence, quality of life, and sexual function, to further refine individualized care pathways for this complex patient population.

## Conclusions

CE remains one of the most complex congenital anomalies encountered in pediatric surgery, requiring meticulous planning and collaboration across multiple disciplines. This case demonstrates that successful outcomes can be achieved through a carefully staged approach emphasizing early stabilization, orthopedic facilitation of closure, and biologically reinforced reconstruction. The evolution of surgical techniques has transformed CE from a fatal malformation into a surgically correctable condition with excellent survival and promising long-term function. Ongoing research and refinement of reconstructive strategies will continue to enhance outcomes for this challenging yet increasingly treatable anomaly.
